# Non-invasive monitoring solutions in the second trimester of pregnancy

**DOI:** 10.25122/jml-2025-0106

**Published:** 2025-08

**Authors:** Madalina Piron-Dumitrascu, Dragos Cretoiu, Valentin Nicolae Varlas, Nicolae Suciu

**Affiliations:** 1 Department of Obstetrics and Gynecology Polizu Clinical Hospital, Alessandrescu-Rusescu National Institute for Mother and Child Health, Bucharest, Romania; 2 Department of Obstetrics and Gynecology, Carol Davila University of Medicine and Pharmacy, Bucharest, Romania; 3 Department of Medical Genetics, Carol Davila University of Medicine and Pharmacy, Bucharest, Romania; 4 Fetal Medicine Excellence Research Center, Alessandrescu-Rusescu National Institute for Mother and Child Health, Bucharest, Romania; 5 Department of Obstetrics and Gynecology, Filantropia Clinical Hospital, Bucharest, Romania

**Keywords:** neonatal outcomes, second trimester of pregnancy, high-risk pregnancy, non-invasive monitoring, fetal and maternal surveillance

## Abstract

Simple, non-invasive, and affordable ambulatory fetal monitoring methods have been integrated into routine prenatal care, with the potential to enhance maternal-fetal health surveillance. Although conventional prenatal care is the basis of pregnancy monitoring, more and more studies are presenting complementary approaches that aim to identify early, potentially pathological changes in fetal status. The use of portable devices, such as a handheld fetal Doppler (for at-home detection of fetal heartbeats) and a pulse oximeter (for maternal heart rate assessment), has been proposed as additional tools in the context of pregnancy monitoring. These devices may influence patients’ behavior regarding seeking medical care and using health services. Access to instruments that allow minimal monitoring at home for pregnant women could facilitate the earlier identification of possible changes, especially in situations where access to direct medical consultations is delayed or restricted. The ease of use and availability of these devices in the broader market raise the question of their integration into a standardized prenatal monitoring framework. This study investigated the feasibility and patients’ perceptions of using handheld Dopplers during pregnancy. An observational analytical study was conducted between January 2019 and December 2023 at the Polizu Clinical Hospital in Bucharest, involving 1,127 pregnant women who met the inclusion criteria (gestational age between 14 and 27 weeks + 6 days and absence of major psychiatric disorders). Of these, 101 women completed a questionnaire regarding fetal monitoring in the second trimester. Responses were analyzed with a focus on the perceived usefulness of the handheld Doppler at home. The majority of participants (79.2%) considered the device helpful, 76.2% reported that it provided them with peace of mind, and 22.8% noted that it led to greater involvement from their partner or family. These findings demonstrate the good acceptability of the tested device, especially among pregnant women in their first pregnancy or with a perceived increased risk, and support the opportunity to integrate this type of monitoring into current obstetric practice. Furthermore, ambulatory and home fetal monitoring solutions provide valuable support in the management of modern pregnancies, but they cannot replace clinical assessment and specialist supervision.

## INTRODUCTION

Pregnancy is a special stage in a woman’s life, marked by profound physiological, emotional, and social transformations. Despite substantial advances in maternal-fetal medicine, which have led to a significant decrease in maternal and neonatal mortality, adverse neonatal outcomes, such as intrauterine fetal death, intrauterine growth restriction, preterm birth, and neonatal distress, continue to represent a challenge in global obstetric practice [1]. These complications occur in various settings, regardless of the level of health system resources, and can occur even in the presence of standardized clinical monitoring [2]. In this context, there is a growing interest in optimizing prenatal care by complementing conventional medical surveillance with complementary, accessible, and patient-centered solutions.

Ambulatory fetal monitoring, using portable devices such as handheld Dopplers, offers the possibility of performing more frequent assessments of fetal well-being in a non-invasive and easy way [3]. These tools can be used both during specialist consultations and in community centers or, under guidance, at home, contributing to strengthening the pregnant woman's relationship with her own pregnancy and active involvement in the prenatal surveillance process.

The appropriate use of these technologies can increase maternal awareness and, in certain situations, can signal early possible changes in fetal well-being, facilitating rapid access to further investigations. Additionally, the subjective dimension of this monitoring, through the potential effect of increasing emotional comfort, has begun to be explored in the specialized literature, particularly in relation to high-risk pregnancies [4].

Pregnancy monitoring encompasses a wide range of clinical and supportive strategies aimed at assessing the health of the pregnant woman, fetal growth and development, placental function, and the overall progress of the pregnancy [5].

Fetal surveillance has undergone significant evolution over time. In 1818, Mayor first described fetal heart sounds, which were later auscultated using the Pinard stethoscope [6]. The introduction of electronic fetal monitoring in the second half of the 20th century allowed continuous assessment of the fetal heart rate during labor. Later, in the 1970s and 1980s, methods such as cardiotocography (CTG), Doppler velocimetry, and biophysical profiling transformed prenatal surveillance into a specialized diagnostic field [7]. In recent decades, digital and mobile technologies have expanded the possibilities of fetal monitoring outside of health facilities, facilitating outpatient assessments and, in some cases, home monitoring.

Antenatal surveillance protocols are developed based on clinical guidelines and resources available in each setting. Recommendations for the frequency of in-person visits for low-risk pregnancies range from 12 to 14 in the US to 7 to 11 in Europe [8]. The World Health Organization (WHO) recommends a minimum of eight structured prenatal contacts, which include physical assessments and health education components [5]. Between 14 and 28 weeks of gestation, monitoring is typically performed every 4 weeks. However, the maternal mortality rate remains high, which requires increased surveillance in high-risk pregnancies. Risk stratification remains a challenge for healthcare providers.

Knowledge of the patient's obstetric history, comorbidities, and fetal and placental factors is predictive of stillbirth.

Fetal monitoring, as part of prenatal care, aims to identify signs suggestive of fetal damage, which may occur in the context of uteroplacental insufficiency, maternal comorbidities, genetic pathologies, or harmful exposures. The primary goal is to identify risk early, followed by appropriate intervention, to prevent the occurrence of severe complications. This dual function – of detection and prevention – is essential, especially in settings with limited access to emergency obstetric services [1].

The present study, conducted at Polizu Clinical Hospital in Bucharest, aimed to evaluate the perception and emotional impact of using a portable Doppler device for fetal heartbeat monitoring during routine outpatient consultations. The study included participants from the second trimester of pregnancy who underwent fetal heartbeat auscultation using a portable Doppler and maternal heart rate measurement with a digital pulse oximeter. Subsequently, each participant completed a standardized questionnaire that targeted aspects such as the perception of the device's usefulness, ease of use, and the emotional impact of this experience.

## MATERIAL AND METHODS

### Study design and participants' characteristics

The observational analytical study was conducted between January 1, 2019, and December 31, 2023, at the Polizu Clinical Hospital in Bucharest, a level III unit affiliated with the ‘Alessandrescu-Rusescu’ National Institute for Maternal and Child Health. This study was conducted on a group of 101 pregnant women in the second trimester of pregnancy, recruited at the ‘Polizu’ Clinical Hospital in Bucharest. A total of 1,127 patients met the inclusion criteria, such as confirmed intrauterine pregnancy with a gestational age between 14 and 27 weeks and 6 days, the absence of major psychiatric disorders, and the ability to provide informed consent. Exclusion criteria were pregnancies with gestational age < 14 weeks and > 28 weeks, twin pregnancies, and maternal comorbidities.

**Figure 1:**
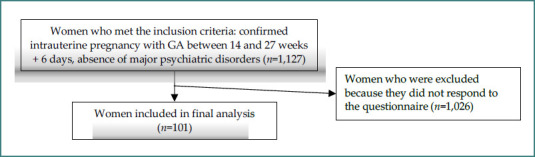
Flow diagram of patient distribution

Of the 1,127 eligible participants identified, 101 women completed a questionnaire regarding the use of portable, handheld Doppler devices for fetal heart rate monitoring. This structured questionnaire collected data on non-invasive methods for monitoring pregnancy, involving the patient, such as the perception of active fetal movements and detection using Doppler, as well as subjective opinions on the usefulness of using the device in a home environment. Participants could select one or more reasons why they considered using the device beneficial, including: personal reassurance, history of miscarriage, or increased partner or family involvement (Figure 1).

Pregnant women with confirmed pregnancy who wanted to respond to the suggested questionnaires were eligible for inclusion in the study. This selection enabled the investigation of pregnancies in the second trimester, confirmed both clinically and paraclinically, to obtain reproducible data on maternal-fetal parameters specific to this trimester. All patients who presented for prenatal consultation at the Polizu Clinical Hospital, in the hospital's polyclinic, part of the National Institute for Maternal and Child Health ‘Alessandrescu-Rusescu’, were evaluated in relation to the inclusion criteria. The selection of participants was carried out according to these pre-established criteria.

### Clinical evaluation and data collection

Fetal heart rate was recorded using two handheld Doppler devices: Fetal Doppler Sonoline C (with 2 MHz probe) and VComin FD200D (also with 2 MHz probe). Maternal heart rate was measured using the iMDK C101A2 pulse oximeter, applied digitally. After recording the parameters, the participants completed a structured questionnaire that included demographic and clinical data, such as current age, height, weight, and known medical conditions. Obstetric history was documented by questions regarding the date of last menstruation, previous births (yes/no and number, if applicable), and previous abortions (yes/ no and number, if applicable). The questionnaire also included questions regarding the participants' experience with the use of handheld Doppler and pulse oximeter devices, including the value of the recorded fetal heart rate and maternal heart rate, as well as opinions regarding the usefulness of these devices for home use, with the possibility of selecting multiple motivations (e.g. personal reassurance, family involvement, history of miscarriage or fetal loss). Additionally, the questionnaire included confidentiality clauses and an informed consent form for voluntary participation.

### Statistical analysis

Data were collected in a Microsoft Excel file and processed using IBM SPSS Statistics for Windows, version 29.0 (Armonk, NY: IBM Corp). The variables analyzed included patient identifier, age, height, weight, body mass index (BMI), presence of pre-existing pathologies, gestational age, number of births, number of abortions, details of the current pregnancy, data related to fetal/ embryonic heart rate recording, fetal heart rate values, maternal heart rate measured with pulse oximeter, as well as perceptions of the usefulness of the devices and benefits identified by the patients.

Continuous variables were expressed by arithmetic mean, median, mode, standard deviation, minimum, and maximum values. Nominal data were presented as absolute frequencies and percentages. Comparison of means for dichotomous variables was performed using the *t*-test for independent samples. Oneway ANOVA analysis was used to compare parameters between multiple groups, followed by multiple comparisons using the Bonferroni post hoc test. The Pearson correlation coefficient was used to evaluate the degree of correlation (r) between the analyzed variables. A statistical significance threshold of *P* < 0.05 was considered relevant.

## RESULTS

In this study, we assessed the use of portable Dopplers for pregnancy monitoring among 101 women aged 16 to 47 years. The participants were categorized based on age, height, weight, BMI, gestational age, comorbidities, fetal heart rate, maternal heart rate, and complications during pregnancy. The analysis of the survey questionnaires generated the study group data, which is detailed in Table 1.

**Table 1:** Clinical characteristics of the study group

Parameter	
Number of cases (*n*)	101
Age (mean ± SD) years	30.82 ± 6.31 (range 16–47)
Height (mean ± SD)	1.67 ± 0.054 (range 1.55–1.8)
Weight (mean ± SD)	80.61 ± 11.97 (range 50–124)
BMI	28.78 ± 3.38 (range 19.3–40)
Comorbidities no yes	90 (89.1%) 11 (10.9%)
Gestational age (mean ± SD)	21.6 ± 3.81 (range 15–28)
Fetal heart rate (mean ± SD)	146.78 ± 19.58 (range 111–182)
Maternal heart rate (mean ± SD)	81.88 ± 15.33 (range 50–123)
Complications during pregnancy (*n*, %) cerclage gestational diabetes preeclampsia preterm prelabor rupture of membranes (PPROM) placenta previa	9 (8.91%) 3 (2.97%) 4 (3.96%) 3 (2.97%) 1 (0.99%)

A fetal heart rate was successfully recorded in 92.1% of women during the second trimester of pregnancy. Overall, 79.2% of participants found the tested device helpful, while 76.2% reported that it provided them with a sense of peace of mind. Additionally, 22.8% indicated that the device encouraged greater involvement from their husband or family, and 3% noted a history of spontaneous abortion.

Bivariate correlation analysis revealed a statistically significant positive correlation between patient age and BMI (r = 0.305, *P* = 0.002). The mean BMI value in women who registered the presence of a fetal heart rate was 28.58, while the mean value for women who did not register the presence of a fetal heart rate was 31.08. According to the *t*-test for independent samples, a statistically significant difference (*P* = 0.044) was found between women who registered a fetal heart rate and those who did not, in terms of BMI value. This suggests that women who recorded the presence of fetal heartbeats had a significantly lower body mass index than women who did not record the presence of fetal heartbeats.

A positive, statistically significant, moderate correlation was observed between the patient's age and the number of pregnancies (r = 0.372; *P* < 0.001). As age increased, the number of pregnancies also increased.

The results of the statistical analysis indicated a statistically significant relationship between the number of births and the effectiveness of the tested device (*P* = 0.001). Furthermore, 19.8% of the women in the second trimester had complications during pregnancy.

To further explore differences in the average number of births across responses to the question *‘How useful do you find the device?’*, we applied the Bonferroni post hoc test. The results obtained revealed that the answer ‘No’ was specific to women who had an average of 0.90 births, while the answer "It reassures me" was specific to women who had a lower average number of births (*n =* 0.35 births). Women who answered ‘Husband/family is more involved’ had an average of 1.67 births.

## DISCUSSION

The analysis of data collected from pregnant women in the second trimester of pregnancy revealed a series of demographic and clinical trends relevant to the use of the portable fetal monitoring device. The mean patient age was approximately 31 years, indicating a balanced distribution within the optimal reproductive period, while the broad age range provided a representative view of the study population’s diversity. Additionally, the values related to weight, height, and BMI indicated an increased prevalence of overweight, a significant aspect in the current obstetric context.

Most women perceive active fetal movements up to 18 weeks of gestation, confirming data from specialized literature [5]. Fetal heart rate was successfully recorded in more than 90% of participants, demonstrating the effectiveness of the device under ambulatory conditions. The mean values of both fetal and maternal heart rates were within physiological limits, and no statistically significant associations were identified between maternal and fetal cardiac parameters.

In recent decades, technological advancements have enabled the decentralization of pregnancy monitoring. Wearable devices, mHealth platforms, and community-based strategies offer the potential to deliver certain components of prenatal care outside the traditional clinical setting [9]. This progress has significant implications for increasing the autonomy of pregnant women and improving equitable access to health services. For example, the use of portable Dopplers to auscultate fetal heartbeats has become a common practice in many primary care settings, providing reassurance for both patients and healthcare professionals [2]. However, significant global disparities persist in the implementation of prenatal monitoring. High-income countries have advanced surveillance technologies, while in resource-limited regions, it is often difficult to provide even basic standards of antenatal care. Early and continuous monitoring is associated with reduced neonatal mortality, but many pregnant women in rural or underserved areas do not receive adequate care [10].

At the same time, educational interventions have become an integral part of modern surveillance. Programs that include fetal movement awareness, the use of mobile applications, or visual biofeedback (such as viewing ultrasound scans with the pregnant woman) are increasingly being used to encourage active engagement. Pilot programs implemented in the Netherlands and the United Kingdom, which involved self-monitoring in high-risk pregnancies, have shown increased maternal satisfaction and a reduction in non-essential clinic visits, without compromising safety [3].

Professional organizations, such as the American College of Obstetricians and Gynecologists (ACOG), the Royal College of Obstetricians and Gynaecologists (RCOG), and the Society for Maternal-Fetal Medicine (SMFM), continue to update protocols and practice bulletins regarding the scheduling and content of prenatal visits. These recommendations include optimal timing for screening for gestational diabetes, anemia, group B Streptococcus infection, and fetal anomalies, thereby integrating monitoring into a structured, staged, and pregnancy-based care model [11]. Telemedicine is also emerging as a valuable complementary tool, especially in the post-pandemic period, facilitating remote consultations and follow-up of pregnant women who cannot access physical health services [12].

A notable aspect is that 79.2% of women perceived the device as useful, primarily attributing its effectiveness to its calming role. This positive psychological effect is important in the prenatal period, contributing to the reduction of anxiety. The fact that the perception of usefulness is not influenced by factors such as age, number of abortions, pregnancies, or gestational age suggests a general applicability of the device, regardless of the obstetric profile of pregnant women. Perceptions of monitoring are also influenced by reproductive history. Women with a history of mis-carriage, preterm birth, or infertility often report intense emotional reactions to fetal monitoring. For these individuals, even routine investigations can trigger anxiety or intrusive thoughts. A 2022 multicenter study showed that women with prior perinatal losses had significantly higher State-Trait Anxiety Inventory (STAI) scores while waiting for ultrasounds, regardless of clinical outcome [4]. In such cases, personalized psychological support and proactive communication are essential to prevent re-traumatization and build emotional resilience.

It is worth noting that pregnancy monitoring is not limited to physiological parameters. In current practice, the psychosocial component is increasingly integrated into antenatal care through screening for affective disorders and psychosocial risk factors, including domestic violence. The correlation between maternal mental health and fetal development is well documented, supporting the need for a multidimensional approach in prenatal care [13].

The analysis of the relationships between variables confirmed the existence of correlations between age and the number of pregnancies or births, which corresponds to clinical logic. Interestingly, women who considered the device useful had, on average, fewer births, which could reflect a higher level of maternal anxiety among primiparous pregnant women, who may perceive the uncertainties of pregnancy more acutely. This hypothesis is supported by the significant differences found between the groups of women who described the device as "reassuring" and those who did not consider it useful.

A statistically significant association was observed between the presence of pre-existing diseases and both higher BMI values and older maternal age. BMI also influenced the feasibility of recording the fetal heart rate, with lower detection rates in women with higher BMI. In contrast, BMI did not affect the actual values of the recorded fetal heart rate, nor was it associated with maternal heart rate or the perception of device usefulness. These findings highlight that BMI has technical implications for signal acquisition but does not influence physiological cardiac parameters or the psychological impact perceived by pregnant women.

Maternal and fetal data collected through portable Dopplers, mobile cardiotocography (CTG), or self-administered kick-chart questionnaires are inherently limited in scope and vulnerable to misinterpretation when assessed outside a clinical context. Several studies have emphasized the psychological impact of home monitoring, noting that ambiguous or difficult-to-interpret results may increase maternal anxiety [14,15]. For example, a qualitative study published in BMC Pregnancy and Childbirth showed that women using self-monitoring tools frequently felt uncertain about the meaning of fetal heart rate values and lacked the knowledge to distinguish between normal and pathological patterns. The study concluded that the use of this method without real-time professional supervision can lead to either overloading of medical services or dangerous delays in seeking medical attention [16].

Another emerging direction is the integration of monitoring data into electronic medical record systems, enabling longitudinal tracking of fetal status and incorporation into maternal early warning systems. Pilot projects are also testing user-friendly interfaces that allow pregnant women to access simplified summaries of fetal parameters, thereby promoting engagement and transparency. In parallel, artificial intelligence–based decision support systems are being developed to synthesize real-time data from CTG, Doppler, and clinical variables, generating personalized predictive scores [17]. Therefore, health systems need to integrate monitoring technologies into the outpatient setting in a responsible manner. The role of these tools is to expand access, stimulate maternal involvement, and support early detection of problems, but always within a structured plan of prenatal check-ups, professional interpretation, and triage protocols. This integrative approach protects against both overmedicalization and underassessment, ensuring that technology facilitates, rather than fragments, the continuum of care [18].

The primary limitation of this study is its relatively small sample size, which may limit the generalizability of the findings. Future prospective research with larger cohorts is needed to identify predictive factors that could guide the selection of patients for fetal monitoring, particularly in high-risk pregnancies. Another limitation is the absence of data on perinatal outcomes and subsequent therapeutic interventions.

## CONCLUSION

The findings demonstrate good acceptability of the tested device, particularly among women in their first pregnancy or those perceiving increased risk, and support its potential integration into routine obstetric practice. Furthermore, ambulatory and home fetal monitoring solutions provide valuable support in the management of modern pregnancies, but they cannot replace clinical assessment and specialist supervision. Misuse of these tools can lead to a false sense of security, delays in diagnosis, or avoidance of life-saving interventions. Their maximum value is achieved not in isolation, but as an integral part of a comprehensive, professionally guided prenatal care program.

## References

[1] Lawn JE, Blencowe H, Waiswa P, Amouzou A, Mathers C, Hogan D (2016). ; Lancet Ending Preventable Stillbirths Series study group; Lancet Stillbirth Epidemiology investigator group. Stillbirths: rates, risk factors, and acceleration towards 2030. Lancet.

[2] Flenady V, Wojcieszek AM, Middleton P, Ellwood D, Erwich JJ, Coory M (2016). ; Lancet Ending Preventable Stillbirths study group; Lancet Stillbirths in High-Income Countries Investigator Group. Stillbirths: recall to action in high-income countries. Lancet.

[3] van den Heuvel JF, Groenhof TK, Veerbeek JH, van Solinge WW, Lely AT, Franx A (2018). eHealth as the next-generation perinatal care: an overview of the literature. J Med Internet Res.

[4] Mainali A, Infanti JJ, Thapa SB, Jacobsen GW, Larose TL (2023). Anxiety and depression in pregnant women who have experienced a previous perinatal loss: a case-cohort study from Scandinavia. BMC Pregnancy Childbirth.

[5] World Health Organization (2016). WHO recommendations on antenatal care for a positive pregnancy experience. https://www.who.int/publications/i/item/9789241549912.

[6] Fowler JR, Mahdy H, Vadakekut ES, Jack BW (2025). Antepartum Care in the Second and Third Trimester. StatPearls.

[7] Dawes GS, Redman CW (1987). Fetal heart rate monitoring. Am J Obstet Gynecol.

[8] Alfirevic Z, Devane D, Gyte GML (2013). Continuous cardiotocography (CTG) as a form of electronic fetal monitoring (EFM) for fetal assessment during labour. Cochrane Database Syst Rev.

[9] Lee SH, Nurmatov UB, Nwaru BI, Mukherjee M, Grant L, Pagliari C (2016). Effectiveness of mHealth interventions for maternal, newborn and child health in low-and middle-income countries: systematic review and meta-analysis. J Glob Health.

[10] Asiimwe JB, Namulema A, Sserwanja Q, Kawuki J, Amperiize M, Amwiine E (2024). Determinants of quality antenatal care use in Kenya: insights from the 2022 Kenya Demographic and Health Survey. PLOS Glob Public Health.

[11] Indications for outpatient antenatal fetal surveillance: ACOG Committee Opinion, Number 828 (2021). Obstet Gynecol.

[12] Thirugnanasundralingam K, Davies-Tuck M, Rolnik DL, Reddy M, Mol BW, Hodges R (2023). Effect of telehealth-integrated antenatal care on pregnancy outcomes in Australia: an interrupted time-series analysis. Lancet Digit Health.

[13] Howard LM, Molyneaux E, Dennis CL, Rochat T, Stein A, Milgrom J (2014). Non-psychotic mental disorders in the perinatal period. Lancet.

[14] Soffer MD, Chen KT (2019). In search of accurate fetal heart rate monitoring mobile applications. Telemed J E Health.

[15] van den Heuvel JFM, Teunis CJ, Franx A, Crombag NMTH, Bekker MN (2020). Home-based telemonitoring versus hospital admission in high-risk pregnancies: a qualitative study on women’s experiences. BMC Pregnancy Childbirth.

[16] Lupton D (2016). The use and value of digital media for information about pregnancy and early motherhood: a focus group study. BMC Pregnancy Childbirth.

[17] Barnova K, Martinek R, Vilimkova Kahankova R, Jaros R, Snasel V, Mirjalili S (2024). Artificial intelligence and machine learning in electronic fetal monitoring. Arch Comput Methods Eng.

[18] Umana OD, Vadakekut ES, Siccardi MA (2025). Antenatal Fetal Surveillance. StatPearls.

